# Repeatability and Reproducibility of Potential Ultrasonographic Bishop Score Parameters

**DOI:** 10.3390/jcm12134492

**Published:** 2023-07-05

**Authors:** Jakub Mlodawski, Marta Mlodawska, Justyna Plusajska, Karolina Detka, Katarzyna Bialek, Grzegorz Swiercz

**Affiliations:** 1 Collegium Medicum, Jan Kochanowski University, 25-369 Kielce, Polandgrzegorzswiercz60@gmail.com (G.S.); 2 Clinic of Obstetrics and Gynaecology, Provincial Combined Hospital in Kielce, 25-736 Kielce, Poland

**Keywords:** induction of labor, elastography, bishop score

## Abstract

Determination of the Bishop score (BS) is a traditional method of assessing the cervix in obstetrics and gynecology. This examination is characterized by subjectivity of assessment and low repeatability. In scientific studies intended to evaluate the results of the procedure based on the initial assessment, it is necessary to find an objective scale based on ultrasonography. We selected five ultrasound parameters, measured with a transvaginal transducer, that are equivalent to the individual BS axes (dilatation assessed in three-dimensional ultrasound (DL), angle of progression (AoP), vagino-cervical angle (VCA), strain elastography using the E-Cervix module, and cervical length (CL)). All selected parameters were characterized by good to excellent repeatability (intraclass correlation coefficient (ICC) = 0.878–0.994) and reproducibility (ICC = 0.826–0.996). Each of the selected parameters significantly correlated with its corresponding BS axis. The highest value of the correlation coefficient was achieved with CL (−0.75) and DL (0.71). Other parameters were characterized by an average to high correlation (AoP and station = 0.69, hardness ratio and consistency = −0.33, position and VCA = −0.38). The best correlation with the sum of the BS points was exhibited by AoP (0.52) and CL (−0.61). The selected ultrasound parameters analogous to the BS axes were characterized by high repeatability and significant correlation with the axes of the original clinical BS. Further research into the predictive properties of a multivariate model based on these parameters is needed.

## 1. Introduction

During pregnancy, the uterine cervix is commonly evaluated through internal examination. This examination helps diagnose the onset of labor, the risk of preterm birth, and the appropriate qualification of patients for the application of cervical ripening agents before the induction of labor (IOL) [1]. In clinical practice, we need a tool that is not only easy to use but also straightforward in interpretation, additionally repeatable, and whose results can be archived in a digital form. The Bishop score (BS) is a widely used point method for cervical assessment. The BS is composed of five elements (five score axes); the total score assumes values from 0 to 13 points [2]. The disadvantage of the BS is its subjectivity of assessment. Furthermore, its reproducibility largely depends on the experience of the examiner [3].

In clinical practice, a BS can determine whether cervical maturity is not overly advanced relative to the duration of pregnancy in patients experiencing abdominal pain during pregnancy. On the other hand, in term gestation, the BS outcome provides information about the chance of the spontaneous onset of labor and its potential duration. For IOL-eligible patients, the BS determines the need for additional methods to promote cervical ripening prior to the start of oxytocin infusion [1]. In most studies using this clinical parameter, an immature cervix is defined as a BS of 6 or less [4]. Therefore, appropriate classification of patients into the <7 and ≥7 BS groups is of the greatest importance. From the point of view of scientific research, an accurate assessment is more important. In the case of an unfavorable cervix, there are many methods of pre-inducing labor available on the market (e.g., prostaglandins, intracervical Foley catheter, hygroscopic dilators) that are designed to increase its favorability [5,6,7]. These methods differ in the mode and potency of action, the route of administration, and the rate of complications observed. Accurate standardization of cervical physiological features prior to their application may, by increasing the repeatability of the examination, be of great use in scientific studies comparing individual methods and allowing the use of a “tailor-made” birth pre-induction method.

The issue we studied has already been addressed in the literature. Some studies have shown better performance of ultrasound-based models compared to the BS [8]. In the described models, ultrasound measurements of the uterine cervix were mainly used, such as the length, dimensions of the cervical funnel, position of the cervix, and assessment of the course of the cervical canal. Other elements used concerned the presenting part of the fetus (angle of progression (AoP), posterior cervical angle (PCA), head-to-peritoneum distance (HPD), head–symphysis distance (HSD), and position of the occiput of the fetus) and its estimated weight. The study also evaluated the anatomical parameters of the pelvis [8,9]. The usefulness of the abovementioned parameters alone and in complex predictive models was investigated. These parameters were assessed using a transvaginal transducer as well as transabdominal and transperitoneal probes.

To decrease the time needed for examination, we assumed the parameters used to be assessable only with the use of the transvaginal transducer (the only method to conduct cervical elastography).

The aim of this study was to assess the repeatability and reproducibility of ultrasound parameters that could potentially be used to calculate the ultrasonographic Bishop score. The second objective was to correlate these parameters with the BS, which was assessed in a clinical study in the same patient.

## 2. Ultrasound Parameters Selected for Examination

The BS consists of five sub-scores assessing the four features of the cervix (length, consistency, dilatation, and position) and the position of the fetal head in relation to the interspinous line. We chose five ultrasound parameters analogous to these five BS axes. Most of these ultrasound measurements are commonly used in clinical practice (e.g., cervical length), whereas the others are mainly applied in scientific research (e.g., cervical strain elastography); additionally, we introduced some of our own modifications. We attached great importance to making the resultant score as simple as possible, using only the intravaginal transducer. Below is a description of the individual selected parameters with their equivalents BS axis.

### 2.1. Cervical Dilatation

**Figure 1:**
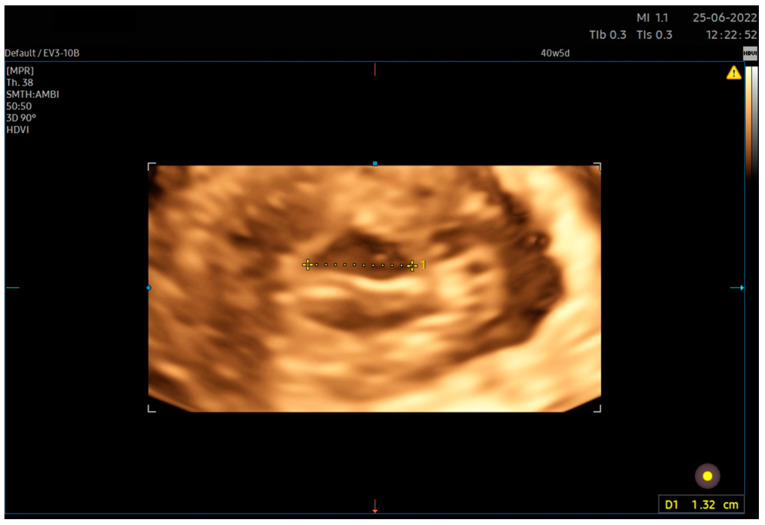
Transvaginal 3D approach for cervical dilatation measurement. We carried out the acquisition of a three-dimensional volume of the cervix. Subsequently, the image was inverted such that the disc of the vaginal portion was in an en face position relative to the examiner. The largest dimension of the external cervix was then measured.

In ultrasound examinations, cervical dilatation is most commonly assessed translabially or transperineally. Alternatively, transabdominal or transvaginal evaluation is possible [10,11,12]. In the literature, both two-dimensional and three-dimensional [10] techniques are described [11]. Both are characterized by a high correlation with the clinical examination (Pearson r = 0.78 (95% CI (confidence interval) 0.72–0.83) for the 3D translabial approach [11], Spearman rho = 0.85 for the 2D vaginal approach [10], and high repeatability (intraclass correlation coefficient (ICC) = 0.85 for the 3D translabial approach and even 0.99 for the 2D translabial approach) [12]. In clinical practice, the ultrasound measurement of dilatation yields an outcome smaller than the actual dilatation assessed in clinical examination. The average difference amounts to 0.9 cm, and the relationship between the clinical and ultrasound results can be expressed by the regression equation y = 1.7 + 0.8x, where x denotes cervical dilatation on ultrasound examination [12]. For the purposes of our analysis, we used 3D transvaginal ultrasound (Figure 1). This is not a form mentioned in the literature because, for technical reasons, it is of little use during labor, when this parameter is most often assessed. During labor with an advanced fetal station and reduced head–perineum distance (HPD), the use of the translabial or transperitoneal technique allows for better volume acquisition owing to a wider imaging angle. Given the group of patients assessed in our study (intrapartum patients were not included in the study), we used volume acquisition using an intravaginal transducer, measuring the largest dimension of the visible volume of the cervix in the area of the external orifice [1].

### 2.2. Position of the Presenting Part of the Fetus

**Figure 2:**
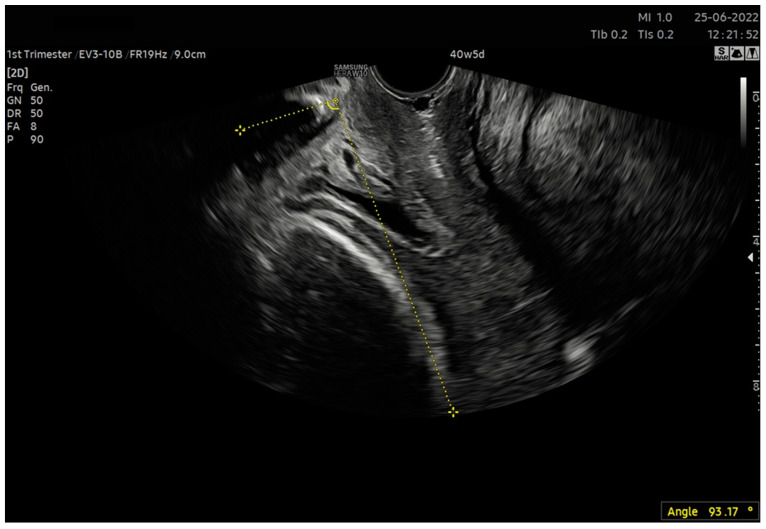
Measurement of the angle of progression (AoP) using the transvaginal transducer. In the examination using a transvaginal probe, the lower edge of the pubic symphysis was visualized. Subsequently, the angle found at the intersection of two sections was traced and calculated: the tangent to the axis of the pubic symphysis and the lowest positioned part of the bony fetal head.

Commonly, this parameter in a clinical study is assessed based on the position of the presenting part of the fetus in relation to the interspinous line. Head–peritoneum distance (HPD), head–symphysis distance (HSD) or angle of progression (AoP) may be the corresponding intrapartum ultrasound parameter [13]. In our analysis, we employed AoP. This is the angle formed between the line parallel to the long axis of the pubic symphysis and the line tangential to the fetal head (Figure 2). Traditionally, this variable is measured translabially. In this situation, the pubic symphysis is closer to the ultrasound transducer than the fetal head. In our study, we used transvaginal measurement, which may be unsuitable in the case of head advancement in the birth canal due to the acoustic shadow cast by the bone parts of the fetal head on the pubic symphysis. For an assessment prior to the preinduction of labor, when the head is not engaged in the birth canal, this approach is sufficient.

Research demonstrates that the value of the AoP is related to the chance of vaginal delivery and correlates significantly negatively with the time to delivery if it is assessed at the moment of admission to the delivery room [14]. AoP assessed before delivery may also be a predictor of the start of a spontaneous vaginal delivery within the next 7 days (OR (odds ratio) = 5.5, 95% CI 1.20–25.24 for AoP ≥ 90°) [15]. AoP assessed at full dilatation during vaginal delivery may predict spontaneous vaginal delivery in singleton, term, and cephalic presenting pregnancies [16].

### 2.3. Cervical Position

Different methods of assessing the position of the cervix are described in the literature. Usually, the uterocervical angle is assessed as the angle between the main body of the uterus and the axis of the cervix. Depending on the position of the axis tangent to the uterus, the following are used: anterior cervical angle (ACA) and posterior cervical angle (PCA). Both variables have been demonstrated to be predictive of IOL success [17,18].

**Figure 3:**
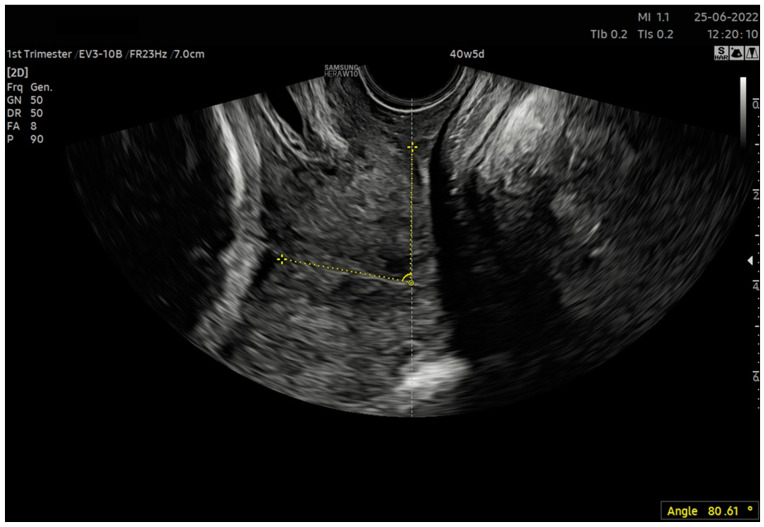
Measurement of the vagino–cervical angle (VCA). Using a transvaginal probe, the cervix was made visible. The angle was delineated between the line tangent to the long axis of the incline and the tangent to the path of the cervical canal.

In our study, we assessed the repeatability of the parameter based on the angle between the vaginal axis and the axis of the cervical canal (VCA—vagino–cervical angle) (Figure 3). In the case of a bent cervical canal, it was a straight line passing through the internal and external cervical os. Despite having not found this type of cervix assessment in the literature, we used it for two reasons. First, in the original score created by Bishop, the position of the cervix was the relationship between the vagina and the cervix [2]; this parameter was also assessed in this way in the clinical evaluation. Second, before delivery, the fetal head, by impinging against the anterior or posterior wall of the uterus and through its “bulging”, modifies its curvature, which may result in an increased correlation between the AoP and ACA or PCA, which is unfavorable from a mathematical modeling perspective in terms of generating predictive models.

### 2.4. Cervical Consistency

**Figure 4:**
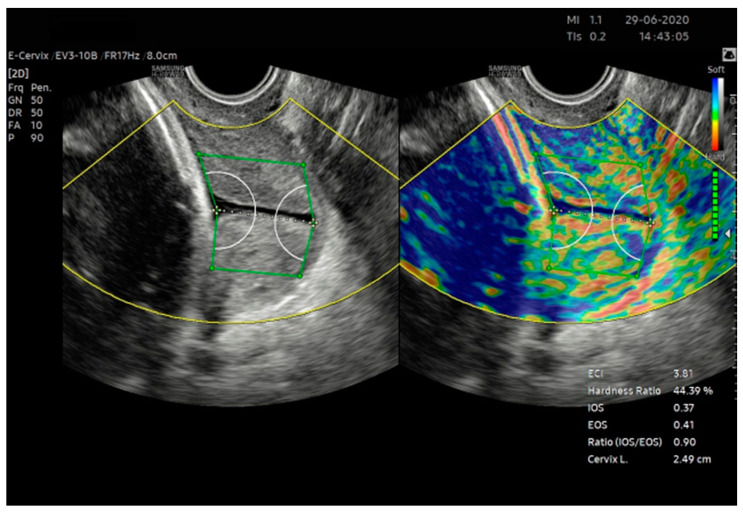
E-Cervix measurement. Examination conducted as per the protocol outlined in Table 2.

In the Bishop score, consistency is assessed on an ordinal scale as a firm, medium, or soft cervix. The elastographic assessment of the cervix appears to be the natural equivalent of consistency in ultrasound scanning. Currently, we have several methods of elastographic examination available, differing in the manner of tissue stimulation and the type of deformation assessed. Shear wave elastography (SWE) allows the quantitative assessment of the wave propagation velocity, which depends on the flexibility of the medium in which it spreads. Studies indicate the predictive capability of cervix SWE for the outcome of IOL [19]. The second method of assessing flexibility in ultrasound examination is strain elastography. The software creates a color elastogram, which can be assessed semi-quantitatively based on color distribution. To date, during this type of imaging, the lack of a quantitative approach to the obtained results has been a problem; elastograms were considered in semi-quantitative scales. This problem is solved in a relatively new approach, which is an examination based on counting the elastogram pixels (E-Cervix ^TM^, Samsung Medison, Seoul, Republic of Korea.) (Figure 4). After deleting the region of interest (ROI) covering the entire cervix, we obtained a quantitative result covering five elastographic parameters and the length of the cervical canal (CL). The obtained parameters, together with the description, are listed in Table 1.

Studies indicate high repeatability and reproducibility of all E-Cervix parameters [20] as well as predictive value in relation to spontaneous vaginal delivery [22] and IOL [23]. The results obtained during the study assessed four absolute elastographic parameters and a single relative parameter (IOS/EOS ratio). In the literature, the EOS parameter is characterized by the lowest repeatability and reproducibility [24]. Due to the proximity of the external cervical os to the transducer, it is the parameter with the greatest sensitivity to the research conditions. HR and ECI are parameters for assessing the entire cervix in elastography. ECI is a measure of cervical heterogeneity, whereas HR is a parameter that informs us of the flexibility of the entire cervix within the ROI on a global scale. E-Cervix is a relatively new method, which is why we used all the parameters unique to this examination for our correlation analysis. We have not yet found an analysis of BS correlation with E-Cervix parameters in the published research.

**Table 2:** Protocol of E-cervix assessment (based on the protocol presented by Hyun-Joo Seol et al. [24] with the authors’ modification in another study [20] and other utilized parameter measurements (CL—cervical length, VCA—vagino–cervical angle, AoP—angle of progression).

Protocol
1. The patient emptied her bladder prior to examination.
2. Image orientation—The apex of the image was displayed at the top of the monitor, and the fetal part was displayed on the left side of the image sector.
3. Activation of the E-cervix program and obtaining of an optimal cervical image— The image plane used for cervical elastography was the same as the one used to measure cervical length (according to the Fetal Medicine Foundation guidelines [25], without applying pressure with the probe to the anterior cervix).
4. Acquisition of cervical strain— After optimal cervical image acquisition, the probe was held still until all motion bars (reliability indicator) turned green (the autofreeze setting for motion bars was used). The patient breathed normally during the acquisition. The image was discarded when active fetal movements occurred during the acquisition, especially fetal limb movement in breech presentation, as it may affect cervical strain.
5. ROI (Region of Interest) caliper placement for strain measurement: (a) Calipers were placed on the grayscale image displayed on the left of the screen, as the elastographic image displayed on the right may be blurred at the margin. (b) By selecting either a 2- or 4-point ROI, a line was drawn along the endocervical canal between the internal and external os of the cervix. If the endocervical line was straight, a 2-point ROI tool was used. With a curved cervix, a 4-point ROI was used to trace the endocervical lining as accurately as possible. (c) After the cervical canal was defined, green points automatically appeared. The points were placed on the four corner edges of the cervix so that the ROI box included the entire cervix area. The entire cervix was included, without adjacent structures such as the bladder or vaginal wall (Figure 4).
6. After calculating the E-Cervix parameters, volumetric image acquisition of the cervix was performed. The 3D image was rotated so that the external os of the cervix was presented en face on the screen; then, we measured the widest dimension of the external cervical os (dilatation—Figure 1). 7. We withdrew the transducer to the area of the vaginal os. 8. The transducer was placed in the vaginal axis; the vagino–cervical angle (VCA) measurement was performed after turning on the “central transducer line” option; the second line was guided tangentially to the axis of the cervical canal. In the case of a bent canal, the line was drawn tangentially to the end part contacting the vagina. We measured the angle formed at the intersection of the abovementioned lines (Figure 3). 9. We measured the AoP with the transducer located near the vaginal opening by drawing two lines—tangent to the long axis of the cross-section through the pubic symphysis and through the bone point of the fetal head being the most advanced in the birth canal (Figure 2).

### 2.5. Effacement

In the Bishop score, effacement is assessed based on the percentage of cervical shortening. Depending on the shortening interval in which the cervix is included, it is possible to obtain from 0 (shortening less than or equal to 30%) to 3 points (shortening ≥ 80%) [2]. Cervical length (CL) is a natural ultrasound equivalent. This variable has long been used in obstetrics; it is mainly applied to assess the risk of preterm birth based on the measurement obtained in the second trimester of pregnancy. There are also reports on the predictive value in relation to the result of IOL [19]. In our study, the cervix was measured in accordance with the recommendations of the Fetal Medicine Foundation in the second trimester of pregnancy [25]. This measurement was obtained in our study automatically with E-Cervix parameters.

## 3. Materials and Methods

We included patients hospitalized over the years 2020–2021 at the Department of Pregnancy Pathology of the Gynecology and Obstetrics Clinic of the Provincial Integrated Hospital in Kielce, Poland. We received approval to examine the patients for research purposes from the bioethics commission at Jan Kochanowski University in Kielce (approval number—55/2019). All methods were performed in accordance with the relevant local regulations and guidelines of the ethical commission. All participants gave informed consent for ultrasonographic examination and participation in the study.

All patients who were included in the study were in their third trimester of pregnancy. We included nulliparous and multiparous women with a singleton pregnancy. Two sonographers, specialists in obstetrics and gynecology, participated in the study. The study patients had a longitudinal lie and cephalic presentation of the fetus, with fetal membranes intact. Due to potential interference with elastographic assessment, patients after cervical procedures (loop electrosurgical excision procedure, conization), after cervical cerclage, or obstetrical pessary placement, with Nabothian cysts and no clear visualization of internal os, were excluded from the study. The examination was performed after the patient emptied her bladder. The whole examination was performed with a transvaginal transducer. All 5 parameters were measured during the examination. The examination protocol is described in Table 2; it was a modified version of the protocols used to assess the repeatability of the E-Cervix examination [20].

To assess repeatability, the operator performed the examination with a transvaginal transducer, consisting of E-Cervix evaluation, dilatation, VCA, and AoP. Then, after removing the transducer from the vagina, the operator performed the examination in accordance with the protocol from the beginning. To assess reproducibility, two operators performed the test immediately after one another, blinded to the other person’s results. Before the ultrasound examination, one of the operators (J.M.) performed the examination of the cervix along with its assessment according to the BS.

### Statistical Analysis

We estimated the minimal sample size based on the table presented by Bujang et al. [26]. Based on the available literature and the preliminary study, we concluded that the value of the intraclass correlation coefficient (ICC) for the null hypothesis should not be less than 0.5 [20,24,27]. With such a null hypothesis, two observations per patient and a power of 90%, the minimum sample size should not be less than 87 subjects. We performed statistical analysis using SPSS 27.0.1.0 software (IBM Company, New York, NY, USA). We characterized the study group using the arithmetic mean and the standard deviation in the case of continuous variables with a distribution close to normal and the median and quartile range for variables with a distribution other than normal. To assess repeatability and reproducibility, we calculated the intraclass correlation coefficient (ICC). The ICC values were interpreted as follows: >0.9—excellent reproducibility, 0.75–0.9—good reproducibility, 0.5–0.75—moderate reproducibility, <0.5—poor reproducibility. Because of the lack of a normal distribution of variables, we used Spearman’s rho coefficient to assess the strength of the correlation. We rejected the null hypothesis in the case of *p* < 0.05. We classified the strength of correlation using J. Guilford’s scale [28].

## 4. Results

The study included 252 pregnant patients between the 27th and 41st weeks of pregnancy. Most patients (59%) had a term pregnancy (37 weeks of pregnancy completed). The demographic characteristics of the study group are shown in Table 3.

We included 140 patients in the group assessing inter-observer variability and 112 patients in the group assessing intra-observer variability (83 for rater A, and 44 for rater B). We present the results of the repeatability and reproducibility assessment in Table 4. Most parameters exhibited excellent repeatability (ECI, IOS, EOS, IOS/EOS, CL, UCA, AoP, dilatation) and good repeatability (HR). In the reproducibility study, all the parameters were characterized by excellent reproducibility (ECI, IOS, EOS, CL, UCA, AoP, dilatation) and good reproducibility (HR, IOS/EOS).

We correlated the individual parameters (mean values from all evaluations) assessed during examination with the parameters assessed in the physical examination. We present the obtained correlations in Table 5. Among the parameters obtained in the E-Cervix examination, the HR parameter exhibited the highest significant correlation; it correlated negatively (rho = −0.33, *p* < 0.05) with cervical consistency assessed by internal examination. None of the elastographic parameters correlated with the cervical summary score of the BS. CL correlated negatively with all the parameters except the cervix position. CL correlated most strongly with its BS counterpart (rho = −0.75, *p* < 0.05); also, VCA correlated most strongly with its BS counterpart (rho = −0.38, *p* < 0.05); however, the correlation achieved an average value. Cervical dilatation measured with ultrasound was strongly positively correlated with dilatation in palpation examination (rho = 0.71, *p* < 0.05). The total score on the Bishop scale showed the strongest correlation with CL (rho = −0.61, *p* < 0.05).

## 5. Discussion

In our study, we demonstrated that the ultrasound parameters we selected and assessed in a pregnant woman in her third trimester, intended to reflect the individual axes of the Bishop scale, exhibit high reproducibility and repeatability. Moreover, all parameters correlated with their respective counterparts on the Bishop scale.

The BS is commonly applied in clinical settings, as the assessment of the cervix using this score is rapid, and the clinician does not need any additional equipment to perform the examination. This scoring system is known globally and has predictive capabilities documented in the literature. Research indicates that a higher BS score increases the chance of vaginal delivery during IOL. Depending on the cutoff point, the OR ranged from 1.98 (95% CI: 1.58–2.48) to 5.48 (95% CI: 1.67–17.96) [29]. Clinical research indicates a negative correlation between all the BS axes and the time of delivery during IOL, although to varying degrees. This correlation fits into the range from low to high, with the lowest correlation exhibited by the position of the cervix (−0.28) and the highest one by dilatation (−0.59). The entire predictive model, consisting of all five parameters, also assumes a correlation value of −0.59 (*p* < 0.05) [30]. A high BS (≥ 6 or ≥ 5 points, depending on the studies included in the meta-analysis) is also one of the factors associated with an increased chance of vaginal delivery after cesarean section (VBAC) (OR = 3.77; 95% CI, 2.17–6.53) [31].

In our view, in daily practice, the BS is a tool sufficient for the correct qualification of patients for the application of various methods increasing cervical favorability (misoprostol, dinoprostone, Foley catheter, etc.). However, in clinical trial conditions, a more sophisticated tool is needed, especially when the trials are conducted in a multicenter setting. The BS is characterized by relatively low repeatability and may be significantly different, depending on the selected pair of researchers. The compatibility of the cervix assessment according to the BS is also related to the experience of the examiner. In one study, the consistency of the results between the examining physicians, depending on the selected pair of physicians, was in the range of ICC = 0.29–0.7 and was much lower for individual BS axes (ICC = 0.06 for position, 0.16 for dilatation) [3]. Slightly better results were observed when the examining physicians were to qualify the cervix as favorable/unfavorable; however, the ICC was still highly variable and ranged from poor to good reproducibility (ICC = 0.46–0.64 depending on the examining pair) [3]. Similar results were obtained in other studies [32]. The number of scientific works assessing the BS in terms of its quality and repeatability, as a measuring instrument available in the literature, is limited. On the other hand, there is an abundance of studies comparing the effects of various procedures based on the initial BS (e.g., [7,33]). In connection with the abovementioned data, creating a high repeatability scoring system is necessary. The parameters of the new system should be preferably related to their equivalents according to the BS.

Previous attempts at using single-factor models based on ultrasound examination have not produced breakthrough results. A meta-analysis from 2015 demonstrated that the assessment of cervix measurement, as the only parameter in transvaginal ultrasound, has no advantage over digital examination, although both methods may be used complementarily [34].

Studies of complex ultrasound models are available in the literature. The transvaginal ultrasound (TVS) cervical score, described by Bajpali et al., was based on the cervix length, dimensions of the cervical funnel, position of the cervix in qualitative assessment, and the distance from the presenting part to the external os. In this study, the created model had greater sensitivity and specificity of effective IOL prediction compared with the BS (sensitivity 77% vs. 65%, specificity 93% vs. 86%) [8]. Several other models that were based on ultrasound parameters, such as estimated fetal weight, cervical length, PCA, AoP, head-to-perineum distance, and fetal occiput position, have been described in the literature with varying reported results [9,35].

In our study, we created a possible prototype of a multivariate score. Simplicity of use was the main goal, which is why we selected parameters measured only with a transvaginal transducer to also limit the quantity of equipment used. The score displayed good correlation parameters with the original BS as well as high repeatability of all parameters. Our analysis is only an introduction to the development of such a score. For it to find application in clinical settings, prospective studies are necessary to assess its predictive abilities, correlations of individual components with clinical outcomes, normalization of variables, determination of specific ranges to assign point scores, creation of centile grids and weights for individual parameters, and validation in other centers. However, we believe that this work will contribute to the popularization of a trend in scientific research aimed at creating a new multivariate measurement tool analogous to the BS, which will be based on ultrasound parameters and might find application in scientific research. The ability to digitally represent the score results in the form of numbers and images also opens the door to the application of artificial intelligence and machine learning methods as well as creating predictive models based on these mathematical methods.

The limitation of this study is its single-center design as well as the hardware requirements: the need to possess a transvaginal transducer with three-dimensional imaging and E-Cervix software for cervical elastographic evaluation, which is currently only available in devices from one manufacturer. However, we believe that other forms of elastography—such as semiquantitative cervical elastography assessment or shear wave elastography (SWE)—could also be used to create the ultrasonographic Bishop score.

A limitation of the study is the inherent subjectivity of ultrasound measurements, although this is certainly lower than the subjectivity of palpation examination. This subjectivity can be reduced by using standardized protocols, as we did in our study. Additionally, baseline parameters such as multiple pregnancy, estimated fetal weight, parity, BMI of women, and histological characteristics of the cervix, may limit the reproducibility of results beyond the conditions in which the study was conducted. Another limitation could be the relatively small sample size in our study.

## 6. Conclusions

It is possible to create an ultrasound score analogous to the Bishop score, characterized by good to excellent repeatability and reproducibility. The scale can then be evaluated from a predictive standpoint. The selected parameters have a significant correlation with their BS counterparts. Further studies are needed to evaluate the predictive properties of the score.

## Figures and Tables

**Table 1:** Description of E-Cervix parameters [20,21].

E-Cervix Parameter	Description
ECI (Elasticity Index)	A measure of tissue heterogeneity. This informs us of the average difference in color intensity between neighboring pixels of the elastogram. It adopts values from 0 to 81 (0—low heterogeneity, 81—high heterogeneity)
HR (Hardness Ratio)	The number of red pixels (defined as the top 30% of the color intensity scale) among all of the pixels in the ROI. This value is displayed as a percentage (0%—soft, 100%—hard)
IOS (Internal Os Strain)	Mean strain level of the internal cervical os ROI (0—hard, 1—soft)
EOS (External Os Strain)	Mean strain level of the external cervical os ROI (0—hard, 1—soft)
Ratio (IOS/EOS)	Ratio of internal and external cervical os mean strain
CL (Cervical Length)	Length of the cervical canal

**Table 3:** Demographic characteristics of the sample group (SD—standard deviation, BMI—body mass index, IQR—interquartile range).

Age (Years) [Mean, SD]	29.57 (4.82)
Multipara [n, %]	92 (36.5%)
Gestational age (weeks) [median, IQR]	38 (4.74)
Weight (kg) [median, IQR]	74 (12.51)
BMI (kg/m^2^) [median, IQR]	26.75 (4.78)
Height (meters) [median, IQR]	1.66 (0.06)

**Table 4:** Repeatability and reproducibility of particular parameters (ECI—elasticity index, HR—hardness ratio, IOS—internal os strain, EOS—external os strain, CL—cervix length, VCA—vagino-cervical angle, AoP—angle of progression, ICC—intraclass correlation coefficient, CI—confidence index).

	Intra-observer Variability			Inter-observer Variability		
Parameter	ICC	95% CI	*p*	ICC	95% CI	*p*
ECI	0.917	0.883–0.941	<0.001	0.907	0.864–0.936	<0.001
HR	0.878	0.829–0.913	<0.001	0.826	0.747–0.880	<0.001
IOS	0.953	0.934–0.966	<0.001	0.918	0.880–0.943	<0.001
EOS	0.918	0.885–0.941	<0.001	0.918	0.881–0.944	<0.001
IOS/EOS	0.907	0.870–0.934	<0.001	0.851	0.784–0.898	<0.001
CL	0.993	0.990–0.995	<0.001	0.993	0.990–0.995	<0.001
VCA	0.988	0.983–0.991	<0.001	0.987	0.980–0.991	<0.001
AoP	0.983	0.977–0.988	<0.001	0.967	0.952–0.978	<0.001
Dilatation	0.994	0.992–0.996	<0.001	0.996	0.995–0.997	<0.001

**Table 5:** Correlation coefficients (* *p* < 0.05).

Bishop Parameter	ECI	IOS	EOS [Mean]	IOS/EOS	HR	CL	VCA	AoP	USG Dilatation
Dilatation	0.09	−0.09	0.11	0.07	0.11	−0.31 *	−0.05	0.18 *	0.71 *
Effacement	0.16 *	−0.03	0.13 *	0.04	0.02	−0.75 *	0.06	0.37 *	0.35 *
Station	0.13 *	0.01	0.05	0.17 *	0.08	−0.44 *	0.00	0.69 *	0.16 *
Consistency	−0.08	−0.05	−0.22 *	0.13 *	−0.33 *	−0.19 *	0.01	0.10	0.08
Position	0.05	0.01	0.20 *	−0.13 *	0.09	−0.11	−0.38 *	0.09	0.09
Sum	0.12	−0.04	0.09	0.09	−0.03	−0.61 *	−0.13 *	0.52 *	0.42 *

## Data Availability

The data that support the findings of this study are openly available in OSF Storage at DOI 10.17605/OSF.IO/KQ2B3.
